# A Magnanimous Gift

**Published:** 1902-06

**Authors:** 


### A MAGNANIMOUS GIFT.

THE Veterinary Department of the University of Pennsylvania has just been enriched by the gift to its library of the entire collection of veterinary and medical books of the late Dr. Rush Shippen Huidekoper.

Through the well-known generosity of Dr. Thomas B. Rayner, of Chestnut Hill, Philadelphia, this valuable collection has been purchased, and it has been given in the name of Moncure Robinson Rayner, a deceased son and former student of the Veterinary Department, who died while completing a course in that department. But one restriction has been placed upon this valuable collection, which is typical, indeed, of the generous character of the donor, that it shall be free to the veterinary profession for consultation and study. Such gifts to any institution are rare enough, indeed; to veterinary institutions of North America it is the second gift of its kind that we have record of, and the first one to be given by a veterinarian. The profession as a whole will be profoundly thankful for this most liberal gift, and the future profession ever grateful for the special privileges it will confer.

